# Effect of bleaching and re-polishing on saffron, turmeric, and black pepper stain removal from anterior composite veneering material

**DOI:** 10.34172/joddd.025.41975

**Published:** 2025-06-30

**Authors:** Fathemeh Dabaghi Tabriz, Katayoun Katebi, Seyedeh Neda Bahiji, Soodabeh Kimyai

**Affiliations:** ^1^Department of Esthetic and Restorative Dentistry, Faculty of Dentistry, Tabriz University of Medical Sciences, Tabriz, Iran; ^2^Department of Oral and Maxillofacial Medicine, Faculty of Dentistry, Tabriz University of Medical Sciences, Tabriz, Iran

**Keywords:** Bleaching, Color stability, Composite resin, Polishing

## Abstract

**Background.:**

This in vitro experimental study evaluated the color stability and effect of bleaching and re-polishing Gaenial, nanohybrid veneering composite resin material, following immersion in saffron, turmeric, and black pepper solutions.

**Methods.:**

Sixty disk-shaped composite resin samples were prepared and randomly assigned to four groups (n=15). The samples were immersed in saffron, turmeric, and black pepper solutions for seven days. Color parameters were assessed using a spectrophotometer before immersion and after seven days. Following immersion, one side of each sample underwent a bleaching treatment, while the opposite side was re-polished. Two-way ANOVA and independent t-test were used to evaluate the effectiveness of the bleaching and re-polishing procedures to remove stains from the composite resin samples. Data analysis was conducted using SPSS 17.

**Results.:**

The groups exhibited statistically significant differences (*P*<0.001). Turmeric solution showed the highest discoloration compared to the other two solutions, followed by saffron, with black pepper demonstrating the least stainability. In the saffron group, no statistically significant difference was observed between the bleaching and re-polishing treatments (*P*=0.06). Conversely, in the turmeric and black pepper groups, there were statistically significant differences between bleaching and re-polishing (*P*<0.001). In the control group, no statistically significant difference was found between the bleaching and re-polishing treatments (*P*=0.378).

**Conclusion.:**

The effectiveness of bleaching and re-polishing varied across the groups. In both the turmeric and black pepper groups, bleaching was more effective in reducing discoloration than re-polishing. Conversely, no statistically significant difference was found between bleaching and re-polishing treatments in the saffron group.

## Introduction

 The advancements in cosmetic dentistry have made it possible to repair damaged and broken teeth using tooth-colored restorative materials.^1^ A key advantage of composite resin materials is their matching color with natural tooth texture, along with their excellent polishability.^2^ However, a persistent challenge remains; discoloration of these restorations, especially when they are exposed to the oral environment over time.^3^ The staining of composite restorations can be categorized into three types: external discoloration, caused by plaque buildup and superficial stains (extrinsic stains); surface or subsurface color change due to minor infiltration of staining agents; and intrinsic discoloration due to physicochemical reactions within the bulk of the restoration.^4^ Addressing these types of discoloration is crucial for maintaining the aesthetic quality of composite restorations over their lifespan. The sensitivity to staining is related to the composition and structure of the resin matrix, along with the properties and size of the filler particles.^5^ Studies have indicated that nano-filled composite resins have shown favorable outcomes.

 Polishing can effectively remove surface stains from heavily stained composite resins; however, removing a surface layer of the composite material has the drawback of removing a surface layer.^4^ Bleaching is an alternative and less invasive method for restoring the original color of stained composite resins. This approach involves a chemical (oxidative) reaction that occurs on the surface of either the tooth or the composite resin. Bleaching effectively targets and eliminates intrinsic and extrinsic pigments in the dental tissues or the composite resin.^6^

 Many research studies have examined the impact of various beverages and substances on the color stability of dental composite resins. Studies have consistently shown that beverages like coffee, tea, soft drinks, and fruit juice can significantly affect the color of these materials.^7^ A study investigating the staining effects of saffron, turmeric, tamarind, and paprika on two types of nano-hybrid composite resins and an indirect base resin highlighted that turmeric exhibited the most significant staining potential among these spices.^5^ Additionally, Usha et al^7^ concluded that both nano-hybrid and micro-hybrid composite resins are particularly susceptible to discoloration from food coloring agents, with turmeric being a notable culprit. In another investigation, Bindal et al.^1^ analyzed the effects of coffee, turmeric, and chewing tobacco on composite resins and glass ionomers. They found chewing tobacco had a more pronounced staining effect than coffee and turmeric. Interestingly, coffee was found to have a greater pigmentation effect between coffee and turmeric.

 Saffron, turmeric, and black pepper are widely used spices in Asian countries, including Iran.^6,7^ While existing research has predominantly focused on the staining effects of beverages like tea and coffee, the impact of food colorants from spices on dental materials requires further investigation. Tooth whitening and re-polishing techniques can differ significantly in their efficacy in removing such pigments, warranting a study specifically designed to evaluate these effects.

 Re-polishing can effectively improve the aesthetics of slightly discolored restorations by removing surface stains, prolonging their lifespan. However, some studies indicate that bleaching may pose risks to dental materials that exhibit high wear and degradation. Limited studies have compared the effect of bleaching and re-polishing methods. Since each material behaves differently, this in vitro study aimed to assess the impact of bleaching and re-polishing of nanohybrid veneering composite resin materials (Gaenial, junior enamel) after immersion in three saffron, turmeric, and black pepper solutions.

## Methods

 In this in vitro study, 60 samples of Gaenial nanohybrid composite resin (Junior Enamel, GC, Asia) were prepared as a disk by a plastic mold measuring 2 mm in thickness and 6 mm in diameter.^5^ Samples with fractures, cracks, and surface bubbles were excluded. Each sample was polished to achieve a smooth surface finish.

 The sample size was determined based on the study by Thaliyadeth et al.^5^ Considering the normal distribution of the data with a standard deviation of 1.42, a mean difference of 3 units between the two groups, α = 0.05, and a power of 0.8, 15 samples in each group and 60 composite resin samples were used.

 A mylar tape was placed over the samples, and a glass slab was pressed onto it to establish a uniform height standard. The samples were light-cured using a device with a light intensity of 1200 mW/cm^2^ (Dentamerica, USA) for 40 seconds. The samples were polished for one minute with polishing discs (TOR VM, Ltd, Moscow, Russia). The samples were placed in an ultrasonic bath (Ultrasonic cleaner ME 4.6R, Mettler Electronics Corp., Anaheim, CA, USA) for one minute to remove debris and then air-dried for 10 seconds.^5^

**Figure 1:**
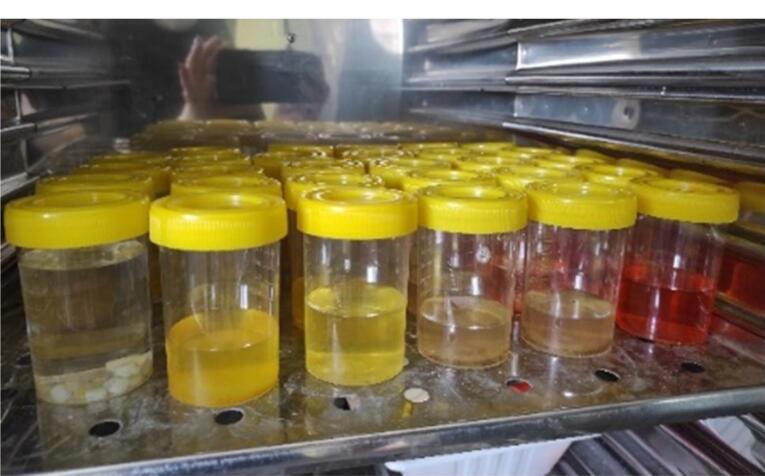
Part of the samples in the incubator

**Figure 2:**
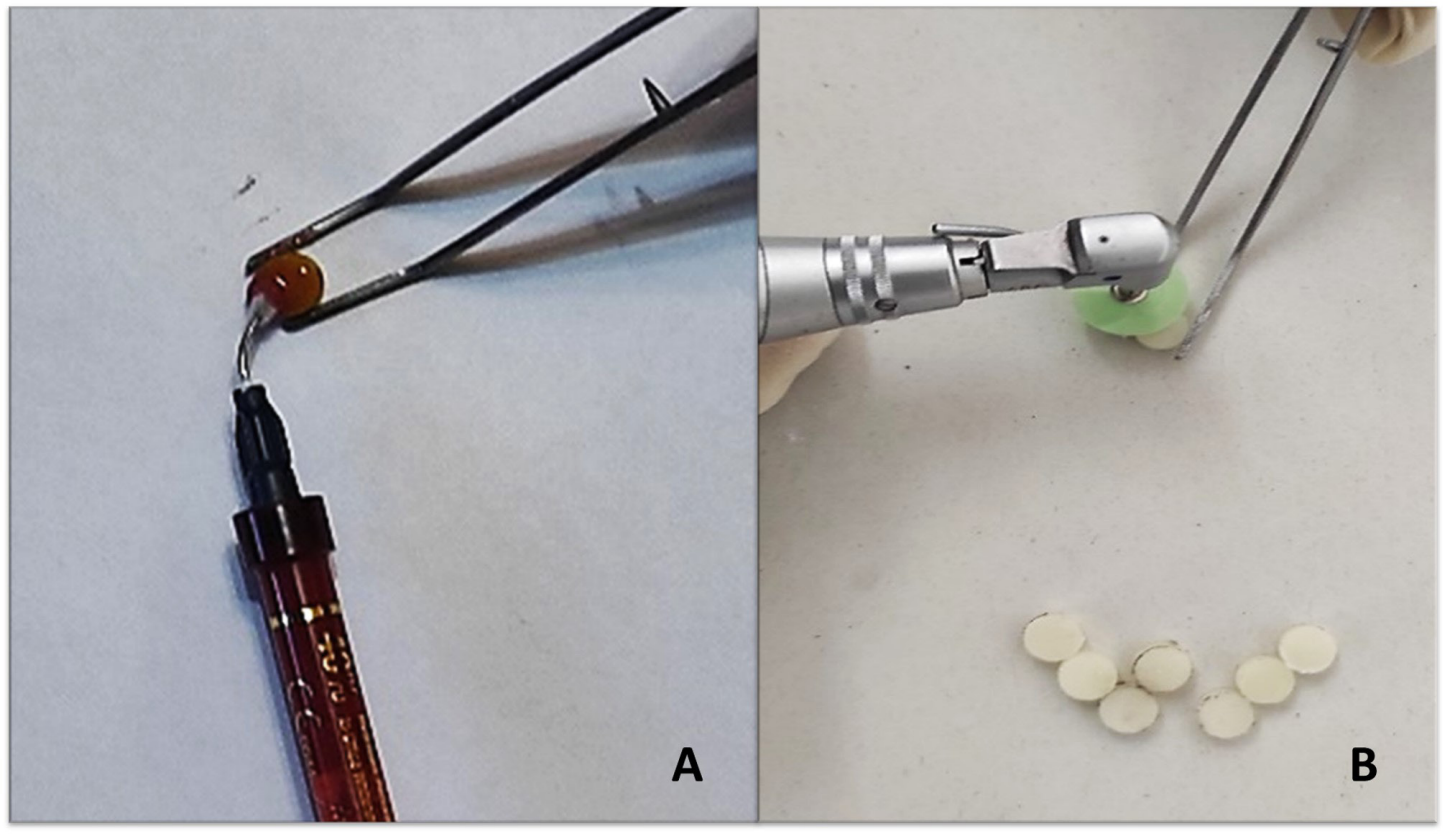
A) Impregnation of composite resin samples with hydrogen peroxide gel. B) Polishing of the samples

 Solutions of saffron (Sahrkhiz, Iran), turmeric powder (Golestan, Iran), and black pepper powder (Golestan, Iran) were created at a concentration of 0.1%. Each spice was added at a ratio of 1 gram per 1000 mL of distilled water (which was heated to 100 °C for 10 minutes and cooled to 37 °C). The samples were randomly divided into two groups by simple randomization. The control group consisted of 15 samples stored in distilled water. Each sample was kept in 20 mL of the desired solution at 37 °C in a dark environment in an incubator (Iran Khodsaz Company, Iran) (Figure 1). The solutions were refreshed every 24 hours. On the seventh day, each sample was subjected to ultrasonic cleaning for one minute using an ultrasonic cleaner (Ultrasonic cleaner ME 4.6R, Mettler Electronics Corp., Anaheim, CA, USA), followed by air drying. ^5^ Before measuring the composite resins’ baseline colors, the spectrophotometer was calibrated using white and green backgrounds under the CIElab system.^5^ One side of the existing samples was polished for 30 seconds with polishing discs (TOR VM, Ltd, Moscow, Russia). The opposite side of the samples underwent a bleaching treatment using 40% hydrogen peroxide (Opalescence Ultra dent, USA) in three stages, each lasting 15 minutes (Figure 2).

 Then, the samples were placed in an ultrasonic bath (Ultrasonic cleaner ME 4.6R, Mettler Electronics Corp., Anaheim, CA, USA) for one minute and dried with air. The samples were assessed with a spectrophotometer on a white background. The color differences between the treated samples and the original composite color were calculated using the following formula:

 ΔE ^*^ = [(ΔL^*^)^2^ + (Δa^*^)^2^ + (Δb^*^)^2^] ^½^

###  Statistical analysis

 The results of descriptive analysis were reported by means and standard deviations. The Kolmogorov-Smirnov test assessed the normality of the data distribution. Differences between materials were evaluated with one-way ANOVA. According to the Levene test results, pairwise group comparisons were assessed with post hoc Tukey tests. A two-way ANOVA and an independent t-test were used to examine the effect of re-polishing and bleaching. The significance level of all tests was considered to be 0.05. Statistical analysis was performed using SPSS 17. The significance level in two-by-two comparisons was considered 0.008 to avoid increasing the maximum acceptable error.

## Results

**Figure 3:**
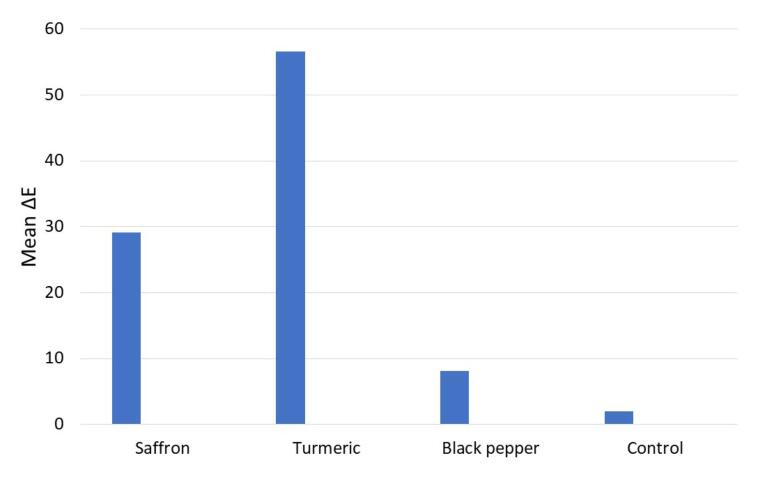
Mean color changes of dye solutions on composite resin samples

Table 1 presents the results of investigating the effect of saffron, turmeric, and black pepper coloring solutions on the color stability of Gaenial Junior Enamel composite resin veneers after seven days. The color change from different dye solutions was statistically significant (*P* < 0.001) (Figure 3). Post hoc Tukey tests were performed to compare the groups (Table 2). The difference between the two groups was statistically significant (*P* < 0.001). The turmeric group exhibited the highest mean color change, while the control group displayed the lowest. The results showed that each dye reacted differently to these treatments (Table 3). To further explore this, independent t-tests were used to compare the mean color changes across different dyes (Table 4).

**Table 1:** Comparison of the effect of dye solutions on the composite resins

**Group**	**Mean ΔE**	**Standard deviation**	**95% Confidence interval **		**Minimum**	**Maximum**	***P*** value^*^
			**Lower band**	**Upper band**			
Saffron	29.17	5.96	25.87	32.47	22.55	44.79	< 0.001
Turmeric	56.63	4.25	54.27	58.98	49.99	66.15	
Black pepper	8.10	1.60	7.22	8.99	6.32	11.37	
Control	1.96	0.53	1.67	2.26	0.79	2.91	

* One-way ANOVA.

**Table 2:** The results of Tukey's post hoc test for two-by-two comparison of dye solution groups

**Group (I)**	**Group (J)**	**Mean Difference (I-J)**	**Standard Error**	***P*** ** value**	**95% Confidence interval **	
					**Lower band**	**Lower band**
Saffron	Turmeric	-27.45	1.37	< 0.001	-31.08	-23.82
	Black pepper	21.07	1.37	< 0.001	17.44	24.70
	Control	27.21	1.37	< 0.001	23.58	30.84
Turmeric	Saffron	27.45	1.37	< 0.001	23.82	31.09
	Black pepper	48.52	1.37	< 0.001	44.89	52.15
	Control	54.66	1.37	< 0.001	51.03	58.29
Black pepper	Saffron	-21.07	1.37	< 0.001	-24.70	-17.44
	Turmeric	-48.52	1.37	< 0.001	-52.15	-44.89
	Control	6.14	1.37	< 0.001	2.51	9.77
Control	Saffron	-27.21	1.37	< 0.001	-30.84	-23.58
	Turmeric	-54.66	1.37	< 0.001	-58.30	-51.03
	Black pepper	-6.14	1.37	< 0.001	-9.77	-2.51

**Table 3:** Comparison of the color change after bleaching and repolishing in different dye groups

**Group**	**Side**	**Mean**	**Standard Deviation**	**95% Confidence interval **		**Minimum**	**Maximum**	***P*** value*		
				**Lower bound**	**Upper bound**			**Group**	**Side**	**Group- Side**
Saffron	Re-polishing	14.78	2.71	13.27	16.28	10.78	20.04	< 0.001	< 0.001	< 0.001
	bleaching	12.76	2.93	11.13	14.38	9.46	21.36			
Turmeric	Re-polishing	37.27	4.571	34.73	39.79	29.38	44.08			
	bleaching	20.37	2.14	19.18	21.56	17.35	24.74			
Black pepper	Re-polishing	3.84	1.25	3.15	4.54	2.30	6.02			
	bleaching	2.22	0.84	1.75	2.68	1.40	4.18			
Control	Re-polishing	2.07	1.17	1.42	2.72	0.64	5.33			
	bleaching	2.45	1.18	1.79	3.11	1.09	1.09			

* Two-way ANOVA.

**Table 4:** Comparison of the effect of re-polishing and bleaching methods in different dye groups

**Group**	**Side**	**Mean**	**Standard** **deviation**	**Mean difference** **(Re-polishing- bleaching)**	**Standard error difference**	**95% Confidence interval **		***P*** value*
						**Lower band**	**Upper band**	
Saffron	Re-polishing	14.77	2.71	2.02	1.03	-0.09	4.13	0.060
	Bleaching	12.75	2.93					
Turmeric	Re-polishing	37.26	4.57	16.90	1.30	14.17	19.61	< 0.001
	Bleaching	20.37	2.14					
Black pepper	Re-polishing	3.84	1.25	1.62	0.39	0.83	2.42	< 0.001
	Bleaching	2.21	0.83					
Control	Re-polishing	2.07	1.16	-0.38	0.43	-1.26	0.49	0.378
	Bleaching	2.45	1.18					

* Independent T test.

**Figure 4:**
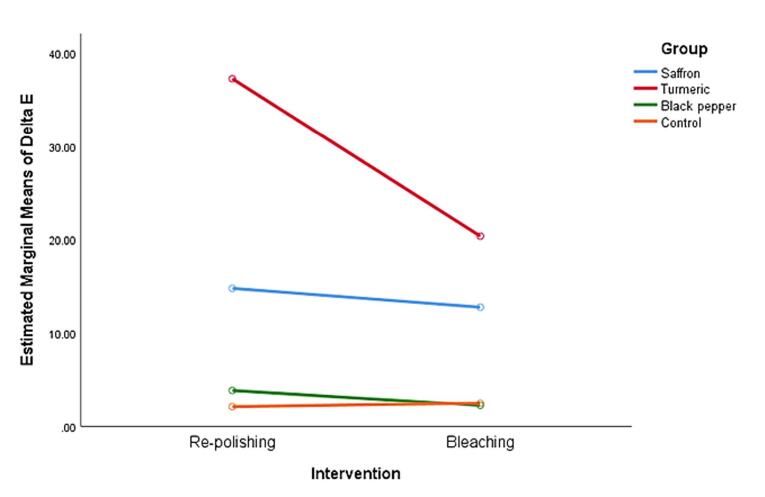
Mean color changes based on different groups and different methods used

 In the saffron group, there was no significant difference between the two methods of re-polishing and bleaching (*P* = 0.06); in the turmeric group, the difference in color change between the two groups of re-polishing and bleaching was significant (*P* < 0.001). In the black pepper group, the difference in color change between the re-polishing and bleaching groups was significant (*P* < 0.001). Still, in the control group, there was no significant difference in color change between the re-polishing and bleaching methods (P = 0.378) (Figure 4).

## Discussion

 Discolored composite resin restorations may be replaced or repaired by finishing, polishing, or bleaching. The interaction between the bleaching agent and restorative material and its role in reversing staining is of clinical interest. Once staining occurs, re-polishing and bleaching procedures can partially or totally remove stains. Most studies have assessed the coloring effect of beverages, and few studies have assessed spices.^8,9^ This study compared the effect of bleaching and re-polishing on the color change of a nanohybrid composite resin.

 All three spices caused a significant color change in the composite samples, with turmeric having the highest effect.Similar to the results of the present study, in the study by Thaliyadeth et al,^5^ all four substances, saffron, turmeric, paprika, and tamarind, caused a significant color change in the composite resins, and all these Indian spices created a stain in both types of composite resins under investigation. Usha et al^7^ compared the effects of food coloring on two types of resin-based composites—micro-hybrid and nanohybrid. The researchers found that all the samples exposed to three different turmeric solutions, and saffron and tandoori powder, showed significant color changes.

 In the study by Yew et al^10^ on the color changes caused by turmeric, paprika, and tamarind spices on resin-based composite resins, similar to our research, they showed that turmeric had the greatest effect on the color of the composite resin. Malhotra et al^11^ reported that the pigmentation effect of turmeric was significantly higher than that of tea and tobacco on all three types of composite resins studied. Turmeric is an ancient spice, and the yellow color of this spice is due to the presence of curcumin.^12^ Turmeric contains large amounts of polyphenols, flavonoids, and ascorbic acid, and studies have shown that high amounts of polyphenols and pigments such as curcumin, desmethoxycurcumin, and bis-desmethoxycurcumin are the reason for the high pigmentation ability of turmeric.^13^ Gupta and Gupta^13^ reported that turmeric had the most significant effect on the coloration of the composite resins studied, followed by coffee, tea, and Pepsi.

 Polyphenols, micron-sized compounds found abundantly in various foods, particularly spices, can cause tooth discoloration. These compounds consist of large chemical chains that contain at least one phenol unit in their structure.^14^ Polyphenolic binary compounds are acidic and release hydrogen ions from their hydroxyl groups. This ionic reaction is responsible for staining composite resins. Research has shown that polyphenols are present in saffron, turmeric, and black pepper, which account for their coloring properties.^15,16^ Turmeric, in particular, has a strong coloring ability due to the presence of curcumin. The conjugated diarylheptanoids found in curcumin are responsible for turmeric’s distinct orange color and higher staining potential.^17^

 This study’s results showed that the choice of treatment method may be more critical for certain dyes, such as turmeric, while for saffron and black pepper, either method may yield similar results.

 Saffron is one of the most expensive spices, and it contains substances such as polyphenol, crocin, crocetin, picrocin, flavonids, and safranal, which is the reason for the coloring of saffron.^18^ In the saffron group, both methods were effective, with no difference in the color change following bleaching and polishing. Jain et al^19^ showed no difference between re-polishing or bleaching procedures in coffee and tea stains. Similar to the results of the present study, the effects of bleaching and polishing depend on the origin of the stains.

 In the turmeric group, bleaching and re-polishing were both effective but significantly different, and re-polishing caused more color changes. This appeared to contradict the results reported by Türkün and Türkün, who reported that polishing was less effective than bleaching because of the deep penetration of staining substances. Such a contradiction may be due to the different types of studied composites. The type of resin matrix has been shown to play an important role in the stain susceptibility of composite resins.^20^

 Black pepper is one of the commonly used spices with antioxidant properties.^21^ In the black pepper group, re-polishing and bleaching were statistically different but not clinically different because color differences of esthetic restorations are considered acceptable up to a DE* of 3.3.^22^ Karademir et al^23^ showed that the effectiveness of re-polishing depends on the material’s staining severity and type,which is consistent with the results of the present study.

 Karadas and Sağsöz^24^ showed that the bleaching agents generated clinically perceptible whitening concerning orange juice, grape juice, tea, and coffee solutions, and their performance was significantly higher compared to re-polishing. The results differ from the present study regarding turmeric, while the results are similar to those of saffron and black pepper. This difference in the results may be due to different staining materials. However, re-polishing is a minimally invasive method to remove external discoloration on restoration surfaces. This method can prevent the replacement of composite resins exposed to external discoloration.^25^

 This in vitro study has several limitations. One is the lack of thermocycling, which could influence the degree of total color change. Another limitation is the short immersion period (one week) in staining solutions. Perhaps prolonged contact may cause further changes in the color index. In addition, the type of resin matrix has been shown to play an important role in the stain susceptibility of composite resin materials. Therefore, several types of composite resin materials should be tested in further research. Also, saliva and intraoral conditions were not simulated, which could affect the results.

## Conclusion

 All three dyes, saffron, turmeric, and black pepper, caused a significant color change in the Gaenial nanohybrid composite resin (Junior Enamel, GC, Asia). The most color-forming effect belonged to the turmeric group. The results showed that the turmeric group exhibited the highest intensity, followed by the saffron and black pepper groups, while the control group displayed the lowest level of color formation. The effect of bleaching and re-polishing on the color difference of different coloring materials was different. In the turmeric and black pepper groups, the effect of re-polishing was reported to be better than the effect of bleaching. There was no significant difference between the bleaching and re-polishing groups in the saffron and control groups.

## Competing Interests

 None.

## Ethical Approval

 The Ethics Committee of Tabriz University of Medical Sciences approved this study (IR.TBZMED.VCR.REC.1400.222).
